# Revision Breast Reconstruction With Biologic or Synthetic Mesh: An Analysis of Postoperative Capsular Contracture Rates

**DOI:** 10.1093/asjof/ojae035

**Published:** 2024-05-16

**Authors:** Jennifer Bai, Sarah Ferenz, Megan Fracol, John Y Kim

## Abstract

**Background:**

Both biologic and synthetic mesh have been found to reduce the risk of capsular contracture, yet there is limited data assessing the use of these scaffold materials in revision breast reconstruction.

**Objectives:**

This investigation sought to assess the ability of either biologic or synthetic mesh to prevent capsular contracture in the revision breast reconstruction population.

**Methods:**

A retrospective chart review was conducted of implant-based revision reconstructions performed by the senior author between 2008 and 2023. Patient demographics and outcomes were assessed, including the incidence of Baker Grade III or IV capsular contractures. Results were compared between biologic and synthetic mesh groups using univariate and multivariate analysis.

**Results:**

Ninety-five breasts underwent revision reconstruction with 90 (94.7%) for correction of malposition, 4 (4.2%) for size change, and 1 (1.1%) for revision after additional oncologic breast surgery. Of these breasts, 26 (27.4%) used biologic mesh and 69 (72.6%) used synthetic mesh. Capsular contracture occurred in 1 (3.8%) biologic mesh breast and 4 (5.8%) synthetic mesh breasts. There was no significant difference in the incidence of capsular contracture between the 2 groups (*P* = 1.000). None of the recorded demographics were risk factors for capsular contracture, including the use of biologic or synthetic mesh (*P* = .801).

**Conclusions:**

Both biologic and synthetic mesh are successful at preventing capsular contracture in patients undergoing implant-based revision reconstruction. This adds to the growing evidence that both scaffold materials can be used in complex revision breast reconstruction to aid in preventing capsular contracture.

**Level of Evidence: 4:**

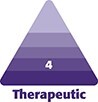

The most common form of breast reconstruction after mastectomy is implant-based reconstruction.^1,2^ Compared with implant-based breast augmentation for aesthetic purposes, these implant-based reconstructions are generally more complex for a multitude of reasons. Reconstruction patients often have a narrow implant pocket and thin tissue overlaying the implant as a result of their mastectomies.^3^ Many patients may also have undergone treatment with radiation or chemotherapy, both of which compromise breast tissue integrity and perfusion.^4,5^ These factors place the breast reconstruction population at an increased risk for complications, which tend to lead to more surgical procedures. Breast reconstruction often requires multiple procedures to achieve satisfactory results.^6-8^ There are several indications for revision breast reconstruction, including implant malposition, capsular contracture, infection, implant rupture, and a desire for implant size change.^9^

Of the complications that occur in breast reconstruction, capsular contracture is one of the most common and has been identified as being one of the leading causes for revision surgery.^10^ In previous breast implant studies, the rates of capsular contracture after primary reconstruction have ranged from 10.6% to 13.7%, with a higher incidence of 10.9% to 25.2% after revision reconstruction.^11,12^ Capsular contracture has been defined as a local complication in which there is tightening of the capsule around the implant. This results in a clinically firm breast, which may cause the patient discomfort or pain.^13,14^ The Baker classification is the most common method to quantify capsular contracture and ranges from Grade I to Grade IV with a higher grade representing more severe contracture.^15^

There have been several theories proposed as to the etiology of capsular contracture, including subclinical infection, biofilm formation, and chronic inflammation, although the development of contracture is likely a multifactorial process.^11^ Definitive management of capsular contracture often requires surgical capsulectomy, but several techniques have been proposed that may reduce the risk and incidence of this complication. These techniques include copious antibiotic irrigation of the implant pocket, submuscular placement of the implant, and the use of textured implants.^16-18^

When addressing capsular contracture in reconstruction patients, it is also important to discuss the use of biologic acellular dermal matrix (ADM) and synthetic mesh. These internal support materials are increasingly being used in secondary revision reconstruction cases for their ability to improve outcomes with regards to capsular contracture, implant malposition, and synmastia.^19^ The use of internal tissue support has been shown, both clinically and through previous research investigations, to significantly improve these complications in the reconstruction population.^20,21^

It has also been hypothesized that either biologic or synthetic mesh may reduce the rates of capsular contracture in breast reconstruction and augmentation patients. However, there is a paucity of data regarding the incidence of capsular contracture after revision reconstruction using either biologic or synthetic mesh with long-term follow-up. Our investigation sought to evaluate how biologic or synthetic mesh use impacted capsular contracture in breast reconstruction over a 14-year period, while also directly comparing outcomes between the 2 materials.

## METHODS

Institutional Review Board approval was obtained for this investigation from the Northwestern University IRB. A retrospective chart review was then performed for revision implant-based breast reconstructions performed by the senior author in which either biologic or synthetic mesh were used. All operations occurred in a hospital setting between June 2008 and May 2023. Patient demographics, operative data, and follow-up information were all assessed. This included patient age, BMI, tobacco history, diabetes history, ASA classification, radiation history, chemotherapy history, laterality of breast reconstruction, implant size, implant surface texture, and use of either biologic or synthetic mesh. The length of follow-up and incidence of postoperative capsular contracture were also recorded. Only patients with a clinically significant Baker Grade III or IV capsular contracture were identified as developing capsular contracture.

### Statistical Analysis

Baseline demographics, the incidence of capsular contracture, and other complications were compared between biologic and synthetic mesh cohorts. A Wilcoxon signed rank test or 2-sided *t* test was used to compare numerical data as appropriate. A Fisher's exact test was used to compare categorical data. A univariate analysis was used to compare demographic information between patients who developed capsular contracture and patients who did not develop capsular contracture. A multivariate logistic regression analysis was then performed to assess potential risk factors for the development of capsular contracture. Statistical significance was set as *P* < .05. Statistical analysis was performed in RStudio version 1.4.1717 (Posit, Boston, MA).^22,23^

### Biologic and Synthetic Mesh

The types of biologic mesh used in this investigation included 2 human-derived ADMs, AlloDerm (LifeCell Corporation, Branchburg, NJ) and FlexHD (Musculoskeletal Transplant Foundation, Edison, NJ). It also included 1 porcine-derived ADM, Fortiva (RTI Surgical, Deerfield, IL).^24-26^

There were also several synthetic meshes that were used in this investigation. GalaFLEX (Galatea Surgical, Inc., Lexington, MA) is a synthetic mesh comprised of high-strength, resorbable poly-4-hydroxybutyrate monofilament fibers.^27,28^ SeriScaffold (Sofregen Medical, Framingham, MA) is a long-term, silk-derived mesh.^29,30^ DuraSorb (Integra LifeSciences, Princeton, NJ) is a resorbable polydioxanone (PDO) mesh with a monofilament and microporous design.^31^

The surgical technique for mesh insertion in these revision reconstructions began by performing a capsulorrhaphy with 2-0 PDS. An inlay pouch was then created with either the biologic or synthetic mesh, and the permanent implant was placed into that pouch of mesh. Steps were also taken during surgery to minimize the risk of capsular contracture, including antibiotic irrigation, a change of gloves, and the use of a Keller funnel.

## RESULTS

Ninety-five breasts in 73 patients underwent revision breast reconstruction with scaffold support. Twenty-six (27.4%) breasts had biologic mesh inserted, and 69 (72.6%) breasts had synthetic mesh inserted. Of the breasts with biologic mesh, 9 (34.6%) used AlloDerm, 16 (61.5%) used FlexHD, and 1 (3.8%) used Fortiva. Of the breasts with synthetic mesh, 43 (62.3%) used GalaFLEX, 20 (29.0%) used SeriScaffold, and 6 (8.7%) used DuraSorb. Of the 95 breasts undergoing revision, 90 (94.7%) were for correction of malposition, 4 (4.2%) were for implant size change, and 1 (1.1%) was revision immediately after complete excision of muscle and capsule overlying implant area because of tumor found on pathology.

There were 73 patients included in this study. The average age of all patients undergoing revision reconstruction was 50.0 years (standard deviation [SD] = 11.3) and average BMI was 26.2 (SD = 6.0). Patients were followed for an average of 4.5 years (mean = 54.3 months, range = 4-150 months, SD = 41.5). Patients with biologic mesh were followed for a significantly longer period of time than those in the synthetic mesh group (91.2 vs 40.5 months, *P* < .001). In terms of relevant medical comorbidities, 2 patients (2.7%) were diabetic and 18 patients (24.7%) were current or former smokers. Three patients (6.7%) were ASA Class I, 32 patients (71.1%) were ASA Class II, and 10 patients (22.2%) were ASA Class III. Twenty-two patients (30.1%) received chemotherapy and 41 patients (56.2%) had undergone bilateral mastectomy. Seventy-one (97.3%) patients underwent subpectoral reconstruction and 2 (2.7%) underwent prepectoral reconstruction. Of the 95 breasts that underwent revision reconstruction, 76 (80%) included biologic mesh at the time of initial implant reconstruction and 19 (20%) did not use either biologic or synthetic mesh at the time of primary reconstruction. Three (3.2%) breasts received neoadjuvant radiation and 10 (10.5%) breasts received postmastectomy radiation. Average preoperative implant size was 536.6 cc (range = 200-960, SD = 149.1) and average implant size after revision was 533.8 cc (range = 125-900, SD = 161.7). Seven (7.6%) breasts had textured implants placed and 85 (92.4%) breasts had smooth implants placed. The average implant size was 533.8 cc (SD = 161.7). Breasts with synthetic mesh had significantly larger implants than biologic mesh breasts (555.7 vs 475.8 cc, respectively, *P* = .013). There were no other significant differences between biologic and synthetic mesh patients for any baseline demographic (Table 1).

**Table 1.** Revision Reconstruction Patient Demographics and Outcomes

	Biologic mesh (*n* = 26)	Synthetic mesh (*n* = 69)	Significance (*P*-value)
Mean age (years)	50.8 (range, 33-72)	49.7 (range, 24-75)	.691
Mean BMI (kg/m^2^)	27.5 (range, 20.5-38.2)	25.7 (range, 17.0-38.2)	.269
Implant volume (cc)	475.8 (range, 325-800)	555.7 (range, 125-900)	.013
History of tobacco use	7 (26.9%)	19 (27.5%)	1.000
Diabetes	2 (7.7%)	1 (1.4%)	.372
ASA class			.513
I	2 (16.7%)	3 (6.4%)	
II	8 (66.7%)	34 (72.3%)	
III	2 (16.7%)	10 (21.3%)	
Neoadjuvant radiation	1 (3.8%)	2 (2.9%)	1.000
Postmastectomy radiation	4 (15.4%)	6 (8.7%)	.567
Chemotherapy	11 (42.3%)	18 (26.1%)	.200
Bilateral mastectomy	17 (65.4%)	45 (65.2%)	1.000
Surface			1.000
Textured	2 (8.0%)	5 (7.2%)	
Smooth	23 (92.0%)	62 (90.0%)	
Length of follow-up (months)	91.2 (range, 24-150)	40.5 (range, 4-139)	**<**.001
Capsular contracture	1 (3.8%)	4 (5.8%)	1.000

The complication profile for this patient population included wound dehiscence (2.1%), seroma requiring drainage (2.1%), hematoma (2.1%), infection (5.3%), and wound necrosis requiring sharp debridement (2.1%). There was no significant difference in the incidence of any complications between the biologic and synthetic mesh groups (Table 2).

**Table 2.** Complication Profile

	Biologic mesh (*n* = 26)	Synthetic mesh (*n* = 69)	*P*-value
Dehiscence	1 (3.8%)	1 (1.4%)	.475
Seroma	1 (3.8%)	1 (1.4%)	.475
Hematoma	0	2 (2.9%)	1.000
Infection	2 (7.7%)	3 (4.3%)	.612
Necrosis	1 (3.8%)	1 (1.4%)	.475
Any complication requiring return to OR	0	2 (2.9%)	1.000

Capsular contracture occurred in 1 (3.8%) biologic mesh breast and 4 (5.8%) synthetic mesh breasts with no significant difference between the 2 groups (*P* = 1.00). The 1 biologic mesh breast used FlexHD and was classified as Baker Grade III. Of the 4 mesh breasts that developed capsular contracture, 2 used SeriScaffold and 2 used GalaFLEX. One SeriScaffold breast was classified as Baker Grade IV, the other 3 mesh breasts were classified as Baker Grade III. In the group of patients who developed capsular contracture, the average age was 63.1 years (SD = 10.9), average BMI was 28.0 (SD = 6.4), and average follow-up was 3 years (mean = 34.2 months, range = 35-113 months, SD = 34.2). Patients who developed capsular contracture were more likely to be older (*P* = .003) and have had a bilateral mastectomy performed (*P* = .048). There were no other significant differences in demographics between the 2 groups (Table 3).

**Table 3.** Capsular Contracture Univariate Analysis

	Capsular contracture (*n* = 5)	No capsular contracture (*n* = 90)	Significance (*P*-value)
Mean age (years)	63.5	49.3	.003
Mean BMI (kg/m^2^)	28.0	26.3	.665
Implant volume (cc)	584.0	531.1	.479
History of tobacco use	1 (20%)	25 (27.8%)	1.000
Diabetes	0	3 (3.3%)	1.000
ASA class			.227
I	1 (50%)	5 (8.8%)	
II	1 (50%)	41 (71.9%)	
III	0	11 (19.3%)	
Neoadjuvant radiation	0	3 (3.3%)	1.000
Postmastectomy radiation	0	10 (11.1%)	1.000
Chemotherapy	0	29 (32.2%)	.319
Bilateral mastectomy	1 (20%)	61 (67.8%)	.048
Surface			1.000
Textured	0	7 (7.8%)	
Smooth	5 (100%)	83 (92.2%)	

Logistic regression analysis was performed to identify risk factors for the development of capsular contracture. There was no significant difference in the risk of developing capsular contracture whether biologic or synthetic mesh was used (*P* = .801). None of the other recorded demographic factors were significant risk factors for developing capsular contracture, including age, BMI, diabetes, history of tobacco use, ASA class, neoadjuvant radiation, postmastectomy radiation, chemotherapy, laterality of mastectomy, implant volume, or implant surface texture (Table 4).

**Table 4.** Risks for Capsular Contracture in Revision Reconstruction

	OR	95% CI	*P*-value
Biologic vs synthetic mesh	0.975	0.797-1.193	.801
Age	1.000	0.993-1.007	.930
BMI	0.994	0.981-1.006	.297
Implant volume	1.000	0.999-1.001	.319
History of tobacco use	0.948	0.828-1.084	.422
Diabetes	1.023	0.706-1.484	.901
ASA class	1.007	0.791-1.282	.954
Postmastectomy radiation	0.965	0.741-1.256	.785
Chemotherapy	0.945	0.811-1.101	.461
Bilateral mastectomy	0.904	0.785-1.040	.154
Surface (textured vs smooth)	1.004	0.704-1.430	.984

OR, odds ratio.

## DISCUSSION

Capsular contracture is one of the most challenging issues that can occur after implant-based breast surgery and is a common reason for reoperation. Capsule formation occurs as a normal foreign body reaction after breast implant placement.^32,33^ The etiology of capsular contracture is multifactorial but involves a chronic inflammatory process that occurs around the breast implant. Factors that can increase inflammation and capsular contracture include bacteria, blood, tissue trauma, and radiation.^34,35^

There has been an increasing use of biologic and synthetic meshes in secondary revisionary breast reconstruction, and common uses for biologic mesh in revision breast reconstruction include capsular contracture, fold malposition, rippling, and synmastia.^36^ A common hypothesis is that biologic and synthetic meshes may be able to reduce rates of capsular contracture.^32,36-38^ There have been some studies evaluating the impact of biologic mesh on capsular contracture rates; however, there is little data on the effect of biologic or synthetic mesh on capsular contracture in revision breast reconstruction.

Our study evaluated how biologic or synthetic mesh use impacted capsular contracture in revision breast reconstruction. Across a 15-year period, our study included 95 breasts that underwent revision reconstruction, and we found that the capsular contracture rate was 3.8% in the biologic mesh group and 5.8% in the synthetic mesh group, which is much lower than the rates of capsular contracture reported in the literature (up to 16.3%) after revision breast reconstruction without the use of biologic mesh.^10^ This suggests that biologic and synthetic mesh may have a protective effect against the development of capsular contracture in a population that is already higher risk given revision breast reconstruction. After regression analysis, biologic and synthetic mesh were not risk factors for the development of capsular contracture and no additional significant risk factors, including radiation, chemotherapy, implant volume, and texture, were identified in this study.

Biologic mesh is thought to be able to alter the biologic response at the interface of the implant and the capsule to help reduce capsular contracture.^32^ With the use of biologic mesh, there has been little capsule formation observed around the portion of the implant in contact with mesh.^33^ Histologic studies have also demonstrated that capsules in implants that use biologic mesh do not exhibit the same degree of inflammatory reaction, myofibroblasts, and fibrosis that is observed with capsules without biologic mesh use.^39^ Nahabedian and Spear hypothesized that the elasticity of biologic mesh can help prevent capsule formation by inhibiting or minimizing the spherical contractile process of capsular contracture.^34^

Our results support the hypothesis that biologic and synthetic mesh can prevent capsular contracture. With the use of biologic mesh, the incidence of capsular contracture has been reported to be 0% to 4% in implant-based breast reconstruction, which is significantly decreased from the rates of capsular contracture in breast reconstruction without biologic mesh which is reported to be 10% to 20%.^32^ Spear et al reported a 2% capsular contracture rate in 50 breasts at 18 months of follow-up with biologic mesh use.^10^ In the Continued Access Reconstruction Revision Expansion trial, Ellsworth et al provided data from a large prospective trial with long-term follow-up of biologic mesh use in breast reconstruction. Capsular contracture rates in the primary reconstruction and revision reconstruction with biologic mesh had lower rates compared with the cohort without biologic mesh. At 5 years of follow-up, the capsular contracture rate in the revision reconstruction with biologic mesh was 1.4% compared with 8.9% in the group without biologic mesh.^19^ Spear et al presented their 5-year experience of biologic mesh use in revision breast reconstruction in which 27.2% of patients had revisional surgery with biologic mesh for capsular contracture and demonstrated that biologic mesh was successful in 95.5% of breasts in the study.^10^

The use of biologic mesh may also result in fewer capsular modifications at the second stage because of better control over pocket dimensions. In a similar manner, the use of biologic mesh as a tissue scaffold may also result in decreased rates of breast revision.^40^ The classic revision rates for tissue expander–based reconstruction without biologic mesh have been reported to be 40% at 7 years using manufacturer-based studies.^41^

Limitations of our study include the retrospective nature of the data and the results from a single surgeon. There were also only 5 patients in our study who developed capsular contracture. This small number limits the univariate and multivariate analyses as it is difficult to assess for risk factors with such a small sample size in the capsular contracture cohort, and there may not be sufficient power to identify true statistical significance. However, we present the data collected over a span of 15 years of follow-up which provides a longer-term assessment of outcomes compared with most existing studies. A larger number of patients and a prospective study design would also provide additional data to strengthen our study's results. Our study includes both biologic and synthetic mesh with approximately 3 quarters of patients having synthetic mesh. The biologic meshes used in this study include Flex HD (61.5%), AlloDerm (34.6%), and Fortiva (3.8%). The synthetic meshes used in this study include GalaFLEX (62.3%), SeriScaffold (29%), and DuraSorb (8.7%). The different types of biologic and synthetic meshes used in this study may introduce some variability into the results. Future studies, including a higher powered study, may be able to assess for differences between these different types of biologic and synthetic mesh as these different types have different inherent biologic properties that can impact capsular contracture and outcomes. Additionally, this study did not look at patients who presented with a postreconstruction capsular contracture that then had mesh inserted. This would be an interesting patient population to assess for recurrence of capsular contracture, and future studies may address the efficacy of mesh in this patient group.

Biologic and synthetic mesh are regarded as beneficial for breast reconstruction and can help facilitate and improve patient outcomes. Biologic and synthetic mesh have been thought to reduce capsular contracture rates, which is supported by the results of our study. Future studies with prospective, randomized trials are needed to further investigate the use of biologic and synthetic mesh in revision breast surgery to further elucidate the benefits of mesh in addressing challenging problems encountered in implant-based breast reconstruction in order to continue to improve patient outcomes.

## CONCLUSIONS

In patients undergoing implant-based revision reconstruction, both biologic and synthetic mesh are effective internal support materials with low rates of capsular contracture. This adds to the growing base of evidence that suggests both materials can be used in complex revision breast reconstruction to prevent the development of capsular contracture. Additionally, there was no significant difference in the incidence of capsular contracture between the 2 materials, suggesting that biologic and synthetic mesh may be similarly effective with respect to decreasing the risk of capsular contracture.
